# Evaluating the Safety of Empiric Tamsulosin to Prevent Postoperative Urinary Retention in a Colorectal Surgery Recovery Pathway

**DOI:** 10.1097/AS9.0000000000000511

**Published:** 2024-11-06

**Authors:** Kurt S. Schultz, Samuel D. Butensky, Thomas R. Hickey, Vanita Ahuja, Melissa F. Perkal, Shilpa S. Murthy, Jaime A. Cavallo, Ira L. Leeds

**Affiliations:** From the * US Department of Veterans Affairs, VA Connecticut Healthcare System, West Haven, CT; † Department of Surgery, Yale School of Medicine, New Haven, CT; ‡ Department of Anesthesiology, Yale School of Medicine, New Haven, CT; § Department of Urology, Yale School of Medicine, New Haven, CT.

## Abstract

**Figure:**
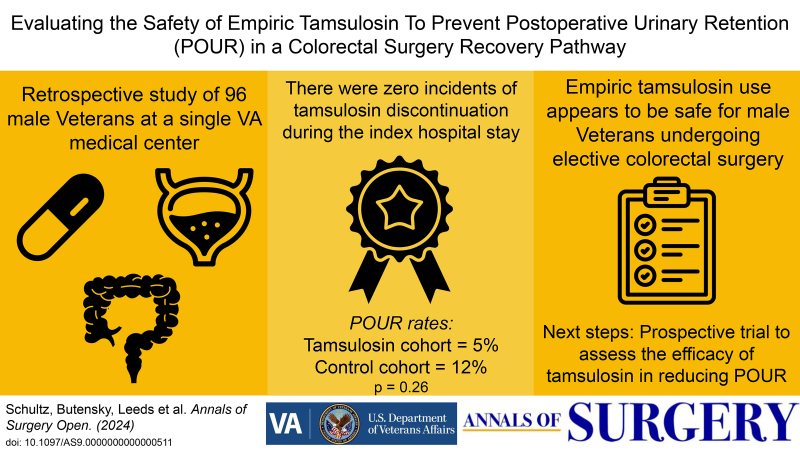

## INTRODUCTION

Postoperative urinary retention (POUR) is a common complication following colorectal surgery.^1^ Risk factors include older age, male sex, and benign prostatic hyperplasia (BPH).^2^ A disproportionate number of veterans have these risk factors compared to the civilian population.^3^ Alpha-1 adrenergic antagonists are the initial medical management of symptomatic BPH, and tamsulosin, which was approved by the Food and Drug Administration in 1997,^4^ is the most prescribed alpha-1 antagonist.

Introducing perioperative tamsulosin into surgery pathways could prevent POUR, decrease catheter-associated urinary tract infection rates, shorten the length of stay (LOS), and reduce emergency room visits or hospital readmissions for catheterization.

The purpose of this study was to evaluate the safety of empiric tamsulosin in male veterans undergoing major colorectal surgery. A secondary goal was to compare the rates of POUR between patients who received tamsulosin and a control cohort who did not receive the drug. The hypothesis was that perioperative tamsulosin use in male veterans, regardless of their age or BPH history, is safe.

## METHODS

This was a retrospective analysis of prospectively collected data of male veteran patients at a single Veterans Affairs (VA) medical center undergoing elective major colorectal surgery, defined as surgery of the colon and rectum via an abdominal incision and excluding anorectal-only approaches, from October 1, 2016, to October 31, 2023. The VA Connecticut Healthcare System’s Institutional Review Board approved this study, and informed consent was waived due to the study’s retrospective nature.

Tamsulosin 0.4 mg nightly was prescribed for 3 days before surgery and continued for 1 week postoperatively. Patients who were already prescribed an alpha-1 adrenergic antagonist for BPH were instructed to continue their medication for the 3 days before surgery. Patients were compared to a control group undergoing elective major colorectal surgery who did not receive tamsulosin perioperatively. Veterans with known contraindications to tamsulosin (ie, allergy, orthostatic hypotension, intolerance to alpha-1 adrenergic antagonists, or concurrent use of a phosphodiesterase-5 inhibitor) were included in the control group. The modified frailty index was used to compare frailty between the 2 groups.^5^ Clinical characteristics related to POUR were collected.

The primary outcome was any incident of tamsulosin discontinuation during the index hospital stay. Secondary outcomes included rates of POUR, rates of urinary tract infections, LOS, return to the operating room within 30 days, 30-day readmissions, and any 30-day Veterans Affairs Surgical Quality Improvement Program-defined complications. POUR was defined as requiring postoperative bladder catheterization. Groups were compared statistically using the Student *t* test for means, Wilcoxon rank-sum test for medians, and Chi-squared test for categorical variables.

## RESULTS

There were 96 male veterans in this study (mean age: 67.6 years, SD: 12.0), with 38 patients in the tamsulosin group and 58 in the control group. Most patients were White (n = 84, 88%) and had a modified frailty index ≥ 1 (n = 71, 74%). The indication for surgery was cancer for 76% (n = 73) of patients. All patients underwent general anesthesia. There were 84 (88%) colon surgeries and 12 (12%) rectal surgeries, with most procedures being performed laparoscopically (n = 77, 80%).

There was a higher percentage of non-White patients (21% [n = 8] vs 5% [n = 3], *P* = 0.017) and less perioperative volume resuscitation in the tamsulosin group compared to the control group (Table 1). There was no significant difference in the proportion of operations involving the pelvis between the 2 groups (10.5% vs 15.5%, *P* = 0.49). In the tamsulosin group, 7 (18.4%) patients were already on BPH medications before surgery (tamsulosin [n = 4], tamsulosin and finasteride [n = 2], and alfuzosin [n = 1]). Two patients included in the control group had a contraindication to tamsulosin, one due to a history of alpha-1 adrenergic antagonist intolerance and the other due to active use of a phosphodiesterase-5 inhibitor.

**TABLE 1.** Demographic and Clinical Characteristics of Male Veterans Undergoing Elective Major Colorectal Surgery at a Single VA Medical Center From 2016 to 2023

	Tamsulosin Group (n = 38)	Control Group (n = 58)	*P*
Age, mean (SD)	68.5 (11.8)	67.0 (12.2)	0.57
BMI, mean (SD)	29.6 (4.8)	28.7 (5.4)	0.39
Race, n (%)			0.017
White	30 (79)	55 (95)	
Non-White	8 (21)	3 (5)	
Ethnicity, n (%)			0.54
Non-Hispanic	33 (87)	51 (88)	
Hispanic	3 (8)	2 (3)	
Unknown/declined to answer	2 (5)	5 (9)	
Modified frailty index, n (%)			0.27
0	13 (34)	12 (21)	
1	15 (39)	25 (43)	
2	5 (13)	16 (28)	
3	4 (11)	4 (7)	
4	1 (3)	0 (0)	
5	0 (0)	1 (2)	
Indication categories, n (%)			0.96
Benign	9 (24)	14 (24)	
Cancer	29 (76)	44 (76)	
Surgical approach, n (%)			0.19
Minimally invasive	33 (87)	44 (76)	
Open	5 (13)	14 (24)	
Procedure categories, n (%)			0.17
Right colectomy	14 (37)	26 (45)	
Left colectomy	20 (53)	20 (34)	
Rectum	4 (11)	8 (14)	
Total colectomy	0 (0)	4 (7)	
Intraoperative IV fluid, mL, median (IQR)	1850 (1500–2100)	2225 (1500–3500)	0.037
Missing, n (%)	0 (0)	2 (3.4)	
PACU IV fluid, mL, median (IQR)	100 (0–300)	275 (100–500)	0.016
Missing, n (%)	1 (2.6)	8 (13.8)	

Percentages may not total 100 due to rounding.

BMI indicates body mass index; IV, intravenous; IQR, interquartile range; PACU, postanesthesia care unit.

There were zero incidents of tamsulosin discontinuation during the index hospital stay for the tamsulosin group. The rate of POUR was 5% in the tamsulosin group (n = 2) compared to 12% in the control group (n = 7, *P* = 0.26). Overall, the average time to POUR was 19.2 hours (range, 9.0–37.3 hours). Other secondary outcomes, including rates of urinary tract infections, LOS, return to the operating room within 30-days, 30-day readmissions, and any 30-day Veterans Affairs Surgical Quality Improvement Program-defined complications, were comparable between the groups (Table 2).

**TABLE 2.** Postoperative Outcomes for Male Veterans Undergoing Elective Major Colorectal Surgery, Dichotomized by Whether Patients Received Empiric Tamsulosin Perioperatively

	Tamsulosin Group (n = 38)	Control Group (n = 58)	*P*
Postoperative urinary retention, n (%)	2 (5)	7 (12)	0.26
Urinary tract infections, n (%)	1 (3)	1 (2)	0.76
Length of stay, d, median (IQR)	5.5 (5.0–8.0)	6.0 (5.0–7.0)	0.78
Return to operating room*, n (%)	0 (0)	3 (5)	0.15
Number of readmissions*, n (%)			0.54
0	37 (97)	55 (95)	
1	1 (3)	3 (5)	
Any 30-d complications^†^, n (%)	7 (18)	11 (19)	0.95

Percentages may not total 100 due to rounding. *Outcome was assessed for occurrence within the first 30 days after the index surgery.

† Defined as any VASQIP-defined complication.

IQR indicates interquartile range; VASQIP, Veterans Affairs Quality Improvement Program.

## DISCUSSION

Most US Veterans are males in their sixth decade of life,^3^ and based on autopsy studies, 50% of this demographic has BPH.^6^ Selective alpha-1 adrenergic antagonists, such as tamsulosin, treat BPH and have fewer side effects than nonselective blockers. Enhanced recovery pathways are recommended for older adults undergoing colorectal surgery^7^ and can decrease LOS,^8^ but historically, these pathways have not included perioperative interventions to address POUR.

In this study, perioperative empiric tamsulosin was safe for male veterans undergoing elective major colorectal surgery, with no incidents of tamsulosin discontinuation. The rate of POUR in the tamsulosin group was 58% less than in the control group, which may allude to a clinically significant finding with future trials powered to evaluate efficacy. Based on this study’s effect size, a sample size of 248 patients per group is required for an adequately powered study to assess the effect of tamsulosin on POUR in patients undergoing colorectal surgery.

A limitation of this study is that patients already being prescribed tamsulosin were grouped with those who were tamsulosin-naive. A second limitation is that the between-group differences in race and volume resuscitation could contribute to confounding. Finally, patients might have experienced known side effects of tamsulosin that were not captured in the VA’s electronic medical record system.^4^ If these side effects did occur, they were presumably not clinically notable, as no patient had their tamsulosin discontinued during their hospital stay.

Male veterans, especially those undergoing colorectal surgery, are at risk for POUR,^1^ which can be a costly complication^9^ and detrimental to a patient’s quality of life.^10^ Adding tamsulosin to established recovery pathways could improve the postoperative recovery experience for veterans. Given the fast onset of action of tamsulosin, its apparent safety profile as demonstrated by this study, and its potential role in preventing POUR, we plan to conduct a randomized trial to assess whether empiric tamsulosin should be added to enhanced recovery pathways for colorectal surgery.

## Acknowledgments

The authors would like to thank Jay Maltz, RN, for assisting in curating data for this study.

Concept and design: KSS, SDB, TRH, VA, MFP, SSM, JAC, ILL. Acquisition of data: KSS, SDB, VA, MFP, ILL. Analysis and interpretation of data: KSS, SDB, TRH, SSM, JAC, ILL. Drafting of the manuscript: KSS. Critical revisions for important intellectual content: SDB, TRH, VA, MFP, SSM, JAC, ILL. Administrative, technical, or logistic support: VA, MFP. Supervision: ILL.
